# The Western Ecotype of the Kazakh Horse: Current Status and Genomic Characterisation

**DOI:** 10.3390/genes17101292

**Published:** 2026-10-16

**Authors:** Kirill Yanin, Rakhim Kanat, Kabyl Zhambakin, Tatyana Ulyanova, Indira Beishova, Alexandr Kovalchuk, Alzhan Shamshidin, Malika Shamekova

**Affiliations:** 1 Tanir Research Laboratory, Almaty 050010, Kazakhstan; k.yanin@ipbb.kz (K.Y.); r.kanat@ipbb.kz (R.K.); k.zhambakin@ipbb.kz (K.Z.); 2 Institute of Plant Biology and Biotechnology, Almaty 050040, Kazakhstan; 3 Zhangir Khan West Kazakhstan Agrarian Technical University, Oral 090009, Kazakhstan; tatyana.poddudinskaya@gmail.com (T.U.); idirabeishova85@gmail.com (I.B.); kovaskst@gmail.com (A.K.); 270180as@gmail.com (A.S.)

**Keywords:** Kazakh horse, western ecotype, breed integrity, introgression, *f*_4_ statistics, SNP array, parentage verification

## Abstract

**Background/Objectives:** The Kazakh horse comprises several locally adapted types and ecotypes of the Eurasian Steppe. The Adai is its western ecotype, kept year-round on desert pasture in Mangystau, Western Kazakhstan, and recognised as a separate breed in 2024 without genetic evaluation. We asked whether that label corresponds to a genetic unit and whether the population has remained closed. **Methods:** We genotyped 533 horses recorded as Adai, 500 traced to five farms in Mangystau, against 988 reference horses of 45 breeds on 36,193 shared autosomal SNPs. Structure was assessed by principal component analysis, pairwise *F*_ST_ with block-jackknife intervals, neighbour-joining trees and cross-validated individual assignment; introgression per animal by an *f*_4_ statistic against an empirical null of 223 reference-steppe horses, with the ancestry scale calibrated on 1800 simulated crosses built from real genotypes; and eight body measurements were modelled within farms. **Results:** The Adai was not separable from the Central Asian steppe complex as a population: pairwise *F*_ST_ was 0.004–0.008 against Tuva, Yili, Sanhe and Mongolian horses, and two Adai farms differed from each other as much as an Adai farm differed from a reference-steppe breed. Individual assignment was nonetheless accurate against those breeds (86–100% pairwise), but the same classifier separated two herds of Adai at 69–85%, so assignment accuracy on thousands of markers tracks marker number rather than breed identity. Against the empirical null, 15.6% of Adai exceeded the 95th percentile and twelve exceeded the maximum. The estimator carries a floor of 3.7 percentage points on an unadmixed Adai; on the calibrated scale, 9.6% of animals reached the ancestry expected from one Thoroughbred great-grandparent and 4.2% that expected from one grandparent (15.8% and 4.6% uncalibrated). Introgression was concentrated: one farm carried 42% of its horses at a great-grandparent equivalent or more against 0–9% at the other four, a difference that survived every analysis accounting for family structure. Ancestry showed no within-farm association with any of eight body measurements, with or without age in the model. **Conclusions:** The Adai is not a differentiated population but part of a continuous steppe complex, and carries undocumented ancestry of Thoroughbred affinity concentrated in particular holdings. As managed, it cannot be certified from a document or a phenotype. Genomic verification at registration is a plausible instrument, but the marker panel it would require was not developed or validated here.

## 1. Introduction

Horse husbandry on the Kazakh steppe is ancient, and in its extensive form has changed little [1]. Herds graze on open pasture for most of the year, mares foal on pasture in spring, and stallions hold harems rather than being assigned to individual matings, a system followed across fifteen years of direct observation in the Aral–Caspian zone [2]. Parentage is therefore never observed and can be inferred only from herd composition. Kazakhstan holds approximately 4.2 million horses and produced 167,400 t of horsemeat in 2023, yet only about 1% of that population is registered as purebred [3]. For the remainder, breed membership is established from provenance and conformation rather than from documented descent. Where breeding is recorded, a breed label is testable from markers: 26 microsatellite loci placed 95% of 306 horses with their own breed and allocated 96% of individuals correctly, including between breeds with a short history of divergence [4], and SNP subsets selected by machine learning reach 93% breed assignment at the most stringent assignment threshold [5]. Whether a label assigned without such records corresponds to anything a marker panel can recover has not been tested for most of the Kazakh horse population.

The western ecotype (Adai) is a saddle-type Kazakh horse of the Mangystau region, between the Caspian and Aral seas, kept year-round on desert pasture where specialised breeds do not survive [6,7]. Until the 2020s, it was classified as an intrabreed type of the Kazakh horse “that underwent historical crossbreeding with riding horses from neighboring regions” [8]. Between November 2022 and January 2024, it was elevated to an independent breed. Its distinctness was judged at a 100 km endurance race under veterinary criteria, reported contemporaneously [9], and the breed certificate was issued by the Ministries of Agriculture and Justice in January 2024 [10]. No genetic evaluation formed part of the assessment.

Differentiation among the recognised Kazakh horse types is far below the level normally found between horse breeds. Pairwise *F*_ST_ among six types and breeds: Zhabe, Adai, Naiman, Kostanay, Kushum and Mugalzhar is 0.0003–0.005 in 2020 horses genotyped on the Equine80k array, with PCA, ADMIXTURE and distance-based analyses all recovering a single cluster and the six proposed to be treated as one breed, the “Kazakh horse” [11]. Zhabe populations behave the same way, with *F*_ST_ among them not exceeding 0.010 apart from one outlying population at 0.027–0.034 [12], and Kushum and Mugalzhar reach only 0.041–0.064 on microsatellites [13]. Maternal markers agree: between-breed mtDNA differentiation is negligible and not statistically significant [8], and Kazakh horses show no significant mtDNA differentiation across the Chinese border, attributed to long-term outcrossing with introduced horses [14]. The nuclear pattern is likewise regional rather than national, with *F*_ST_ among native Xinjiang horse breeds not exceeding 0.01 [15]. For scale, mean *F*_ST_ among seven Spanish Celtic horse breeds is 0.08 [16], and the median between-breed value for horses on SNP data is 0.08–0.12 [17]. Genomic resources for the Kazakh horse are accumulating, including a de novo assembly of the Zhabe type [18], but the largest global comparison to date carried only 27 Adai into a merged panel of 1176 animals on 35,419 SNPs [11,19]. What that work leaves open is whether ancestry from outside the steppe is spread thinly through the population or concentrated in particular herds, which requires ancestry estimated per animal in a sample of documented herd provenance.

Such ancestry has been detected in Kazakh horses but never quantified. Outlier genotypes carrying Thoroughbred ancestry appear in both large SNP studies, placed adjacent to Thoroughbreds in phylogenetic reconstruction and retaining a Thoroughbred component at every value of *K* in ADMIXTURE [11,12]. Every animal in the larger of the two had been “attested as purebred Kazakh horses” before sampling, which the authors read as raising concerns about the “present state of horse breeding control and certification” [11]. The mechanism is documented. Kazakh horses were improved by Thoroughbred, Orlov Trotter and Don stallions in the late twentieth century, and the Adai is one of the two most widespread of the ecological types into which the breed has differentiated [20]. Within the Adai’s own range the record is more specific: more than 400 stallions of specialised meat breeds were brought into the Mangystau and Atyrau regions between 1960 and 1987 to raise carcass weight; the resulting unsystematic crossing is held to have degraded the ecotype to the point of threatening its survival, and such imports were reported to be continuing [6]. The breeding method prescribed for the Adai is nonetheless pure breeding, with improvement by selection within the type rather than by crossing [21]. Work on the population is otherwise dominated by productivity traits such as growth, slaughter yield, meat composition and milk yield under pasture management [7,21,22,23], and its genetic sampling has been thin, at *n* = 10 for mtDNA [8] and 27 of 303 genotyped animals carried into the global comparison [11].

Quantifying recent introgression in this system is constrained by the composition of the available reference panels. Model-based clustering apportions ancestry among the source populations supplied to it, and will attribute foreign ancestry to any population whose own lineage is unrepresented. No publicly available panel contains an unadmixed Western-Kazakh steppe horse. The limitation is not hypothetical here: under supervised ADMIXTURE with the obvious reference groups as sources, a held-out negative control—the Tuva horse, a Siberian steppe breed with no plausible Thoroughbred ancestry—received a non-steppe ancestry estimate indistinguishable from that of the Adai, so that any estimate taken from that model would reflect the incompleteness of the reference panel rather than the ancestry of the sample. The test for introgression was therefore built on the *f*_4_ statistic [24], evaluated against an empirical null distribution measured on reference-steppe horses, which requires no assumption that the reference panel is complete.

The objectives of this study were (i) to position the Adai among 45 reference breeds genotyped on shared autosomal SNPs; (ii) to test whether the breed label corresponds to a genetic unit; (iii) to test for and quantify recent introgression from outside the steppe at the level of the individual animal, under explicit positive and negative controls; (iv) to compare the five sampled farms; and (v) to characterise the relatedness and inbreeding structure of the sampled population. Objectives (ii) and (iii) are independent and carry different consequences for management: a population may be weakly differentiated from its neighbours and still be closed, as local breeds in contact zones commonly are, or well differentiated and still admixed.

## 2. Materials and Methods

### 2.1. Sampling and Body Measurements

Horses recorded as Adai were sampled at five commercial farms in Mangystau region, Western Kazakhstan, on the Mangyshlak peninsula between the Caspian and Aral seas. Farms are reported anonymised as Farm A to Farm E, and the key is held separately. Sampling was non-invasive throughout: hair roots were pulled from the mane by the holding’s own staff during routine handling. Of 570 animals sampled, 533 passed genotyping quality control and 500 of these were assigned to a named farm (Table 1); the 33 horses carrying no farm record were excluded from every analysis except the individual-level tree of Section 3.1, which is drawn on the full merged panel and labelled as such. The sample is predominantly female, as the breeding structure of these holdings implies: 442 mares, 57 males and one animal whose sex was not recorded, with both sexes present at all five farms. Year of birth was recorded for 299 of the 500 and runs from 2002 to 2019 with a median of 2014; it is missing for two-fifths of the sample, which bears on how age can be handled in the trait models (Section 2.5). Body measurements were taken by the holdings under contract: withers height, oblique body length, chest girth and cannon girth in centimetres and live weight in kilograms, available for 499, 499, 489, 496 and 488 animals respectively.

### 2.2. DNA Extraction and Genotyping

Genomic DNA was isolated from hair-root (follicle) samples using the DNA-Extran-2 kit (Syntol, Moscow, Russia) following the manufacturer’s protocol. All samples were genotyped on a custom Equine 80K SNP array built on the Illumina equine design [25], using the iScan system (Illumina, San Diego, CA, USA). Raw intensity data were processed and genotypes called in GenomeStudio v2.0 (Illumina).

### 2.3. Quality Control

Sample-level filtering in PLINK 1.9 [26] at --mind 0.05 removed seven horses, all from the published reference sets, leaving 1514; one of them, Debao-DB17, was contaminated and had acted as an attractor in distance space. A/T and C/G markers were dropped from the Adai data, whose two batches were exported on opposite strands; cross-dataset duplicates were removed at PI_HAT ≥0.85; Hardy–Weinberg was tested within Adai only, since across populations departure from HWE is the structure being measured; and variant QC was applied at --geno 0.05 --maf 0.01. LD pruning was applied where required, at *r*^2^ < 0.2 throughout, and the number of markers retained depends on the window used: pairwise pruning in PLINK over a 50-marker window with step 5 retains 21,649, used for relatedness and for ADMIXTURE; pruning in SNPRelate over a 500 kb window retains 13,598, used for the principal components and the differentiation estimates; and the same SNPRelate procedure over a 50-marker window retains 13,551, used for the assignment test. Each count is given again with its analysis in Section 2.5. Three panels follow from this and are distinguished throughout: 988 reference horses and 533 Adai give the full merged panel of 1521, used only for the individual-level tree of Section 3.1; removing the seven call-rate failures gives the call-rate-filtered panel of 1514; and removing in addition the 33 Adai without farm provenance gives the provenance-filtered panel of 981 reference horses and 500 Adai, 1481 in total, on which every other analysis reported here is run. Section 3.7 reports the main results across all three.

### 2.4. Reference Panel

A total of 988 reference horses of 45 breeds were assembled from three published sources (Table 2). The set was not curated for this study. Our study incorporates every public dataset we could identify that overlaps this array on EquCab2 autosomal markers, taken as a whole; no breed was selected for inclusion and none was dropped, so the composition of the panel reflects what has been deposited for the horse rather than any choice of ours.

Datasets were merged on chr:pos in EquCab2. Only the EVA set required liftOver from EquCab3, and cross-chromosome mappings were discarded. After quality control, this leaves 36,193 autosomal markers shared by all four platforms and an analysis panel of 1481 horses in 50 populations: 45 reference breeds and the five Adai farms. For presentation only, breeds were grouped into six regional groups: Central Asian steppe (10 breeds), Oriental (3), European draft and pony (14), Iberian and Italian (3), European sport and racing (6), and Americas (9); group membership is never an input to the introgression test.

### 2.5. Population-Genetic and Statistical Analysis

Principal component analysis was run in SNPRelate 1.40.0 [29] on the 13,598 LD-pruned markers in the relatedness-pruned sample, obtained by excluding one animal of every pair with KING kinship ≥0.0884 across the whole panel rather than within the Adai alone; this leaves 1121 horses, 207 of them Adai, with all five farms represented (Farm A: 71 of 205, Farm B: 35 of 94, Farm C: 36 of 69, Farm D: 46 of 99, Farm E: 19 of 33). Retention is lowest at the farms with the densest family structure. Ten components were retained. Neighbour-joining trees were fitted to 1 − IBS distances on all autosomal markers using ape 5.8.1 in R 4.4.1 and drawn with ggtree 3.14.0. *F*_ST_ was computed after Weir and Cockerham [30] as the mean of the per-marker estimates, with a standard error from a block jackknife over the 31 autosomes, the same resampling scheme used for the *f*_4_ statistic below; heterozygosity, minor-allele frequency and *F*_IS_ were computed per population. Individual assignment used the frequency-based likelihood method of Paetkau et al. [31] and of Rannala and Mountain [32]: the log-likelihood of each horse’s genotype was evaluated under Hardy–Weinberg from every candidate population’s allele frequencies, estimated with that horse left out and carrying a +1/+2 pseudocount, and the animal assigned to the population of highest likelihood. It was run on 13,551 LD-pruned markers, with the Adai represented by the 207 relatedness-pruned animals so that family structure could not make the breed look recoverable, and with every population subsampled to the smallest (*n* = 15) over 100 draws so that the Adai did not gain from better-estimated frequencies. Three controls were run with the same classifier: two Adai farms against each other, the Adai pool restricted to animals below 5% estimated ancestry, and reference breed pairs from the same published source against pairs from different sources. Tree coherence was taken for each Adai horse as the share of its ten nearest tips that are not Adai, and horses above 50% are termed foreign-embedded. Relatedness among Adai was assessed using KING-robust kinship [33], computed in PLINK 2.0 [26]. Runs of homozygosity were called in PLINK 1.9 [26] with --homozyg-kb 1000, --homozyg-snp 30, --homozyg-density 100, --homozyg-gap 1000, --homozyg-window-snp 30, --homozyg-window-het 1, --homozyg-window-missing 5 and --homozyg-window-threshold 0.05, and *F*_ROH_ computed against 2211.5 Mb of panel-covered autosome. At a mean spacing of about 61 kb, only long runs are detectable, so *F*_ROH_ here is comparable across the panel but is not an absolute value, and cross-breed comparison carries the residual ascertainment of the source arrays even though all horses are reduced to the same 36,193 markers. Introgression was tested with *f*_4_(*i*, *B*; *C*, *D*) [24] for each horse i, where *B* is a Chinese–Mongolian steppe reference of five populations, *C* is Thoroughbred (36), and *D* is heavy draft (100); where i belongs to *B*, it was left out of *B*, and standard errors come from a block jackknife over the 31 autosomes. The statistic was implemented in R rather than in Dsuite [34], which does not return per-individual leave-one-out reference frequencies. B and the empirical null serve different purposes and are not the same set of animals: *B* supplies the reference allele frequencies inside the statistic, while the null is the same statistic evaluated across all 223 reference Central Asian steppe horses of ten breeds, of which the five *B* populations are a subset. Thoroughbred-equivalent ancestry is the *f*_4_-ratio α = *f*_4_(*i*, *B*; *C*, *D*)/*f*_4_(*C*, *B*; *C*, *D*), floored at zero; in the event no Adai required the floor: every one of the 500 returned a positive *f*_4_, while 105 of the 223 reference-steppe horses are negative, as a null centred near zero should be, and the null is used untruncated. No α exceeded one. The jackknife as implemented resamples the numerator against a fixed denominator; resampling both together moves the standard error of α by a median of 1%, by less than 6% in either direction for ninety per cent of the horses, and by at most +16% and −45% at the extremes, so the simpler form is reported and Table S5 gives both per horse. Sensitivity to the choice of *B* was assessed by recomputing α under six definitions of the reference, from a single population to all ten steppe breeds. The ancestry scale itself was calibrated by simulation: 300 pseudo-crosses per ancestry level were built from real genotypes by drawing one allele per marker from each parent, giving F1, first, second, third and fourth backcrosses to the Adai and an Adai × Adai control, with the Thoroughbred donor left out of *C* because the sire of a real crossbred is not in the panel either, and recipients drawn from the 103 Adai below 5% estimated ancestry. The whole cascade was repeated with an Arabian donor to establish what the estimator reports when the true source is a different non-steppe breed. Calibrated ancestry is the inverse of the fitted line, floored at zero, and both scales are reported throughout. Expected F1 heterozygosity for Adai × Thoroughbred was used as a ceiling separating crossbreeding from contamination. Supervised ADMIXTURE 1.3 [35] failed its held-out negative controls, so only unsupervised runs are reported: *K* = 2–6 on the farm panel (1481 horses, 21,649 LD-pruned markers), three replicates per *K* with seeds 1–3 and 10-fold cross-validation, summarised with CLUMPAK [36] and CLUMPP [37]. Body measurements were modelled by ordinary least squares as trait ~ α + farm + sex, one model per trait, with farm as a fixed effect because ancestry is confounded with holding and the two contractor measurement records differ by 3.8 cm in mean withers height; the reported coefficient is therefore the within-farm association. The eight traits are the five measurements of Section 2.1 and the three zootechnical indices derived from them, and *p*-values across the eight were corrected by the Holm procedure. Because year of birth is missing for two-fifths of the sample, age enters as a sensitivity analysis on the 299 animals that have it rather than as a covariate of the primary fit. Relatedness was handled in the same way, by refitting on one animal per family and by permuting α within farm and family, families being the connected components of the first-degree KING graph. The farm comparison was likewise repeated on the relatedness-pruned sample, on one animal per family over 1000 draws, under a linear mixed model with family as a random effect, and under a permutation that reassigns whole families between farms.

## 3. Results

### 3.1. Population Structure and Differentiation

**Figure 1:**
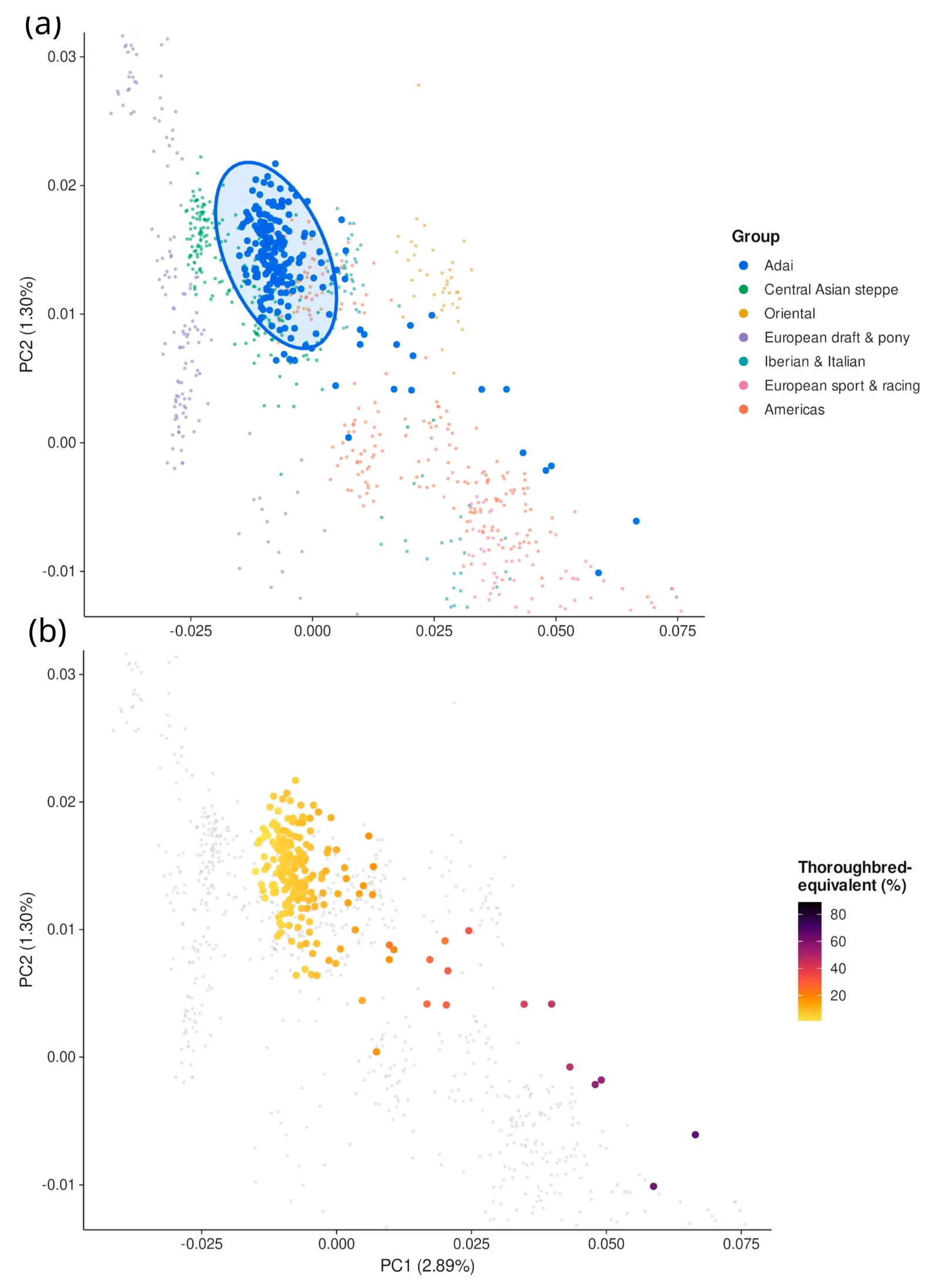
Principal component analysis of the relatedness-pruned panel: 1121 horses of 50 populations on LD-pruned autosomal markers. PC1 and PC2 carry 2.89% and 1.30% of the variance. (**a**) One point per horse, coloured by regional group, with the 95% ellipse of the 207 Adai in blue: the Adai lie inside the Central Asian steppe cloud rather than beside it. (**b**) The same projection with every reference horse in grey and each Adai coloured by its *f*_4_-ratio Thoroughbred-equivalent ancestry (1.4–88.9%); the Adai displaced along PC1 are those carrying the most Thoroughbred ancestry.

**Figure 2:**
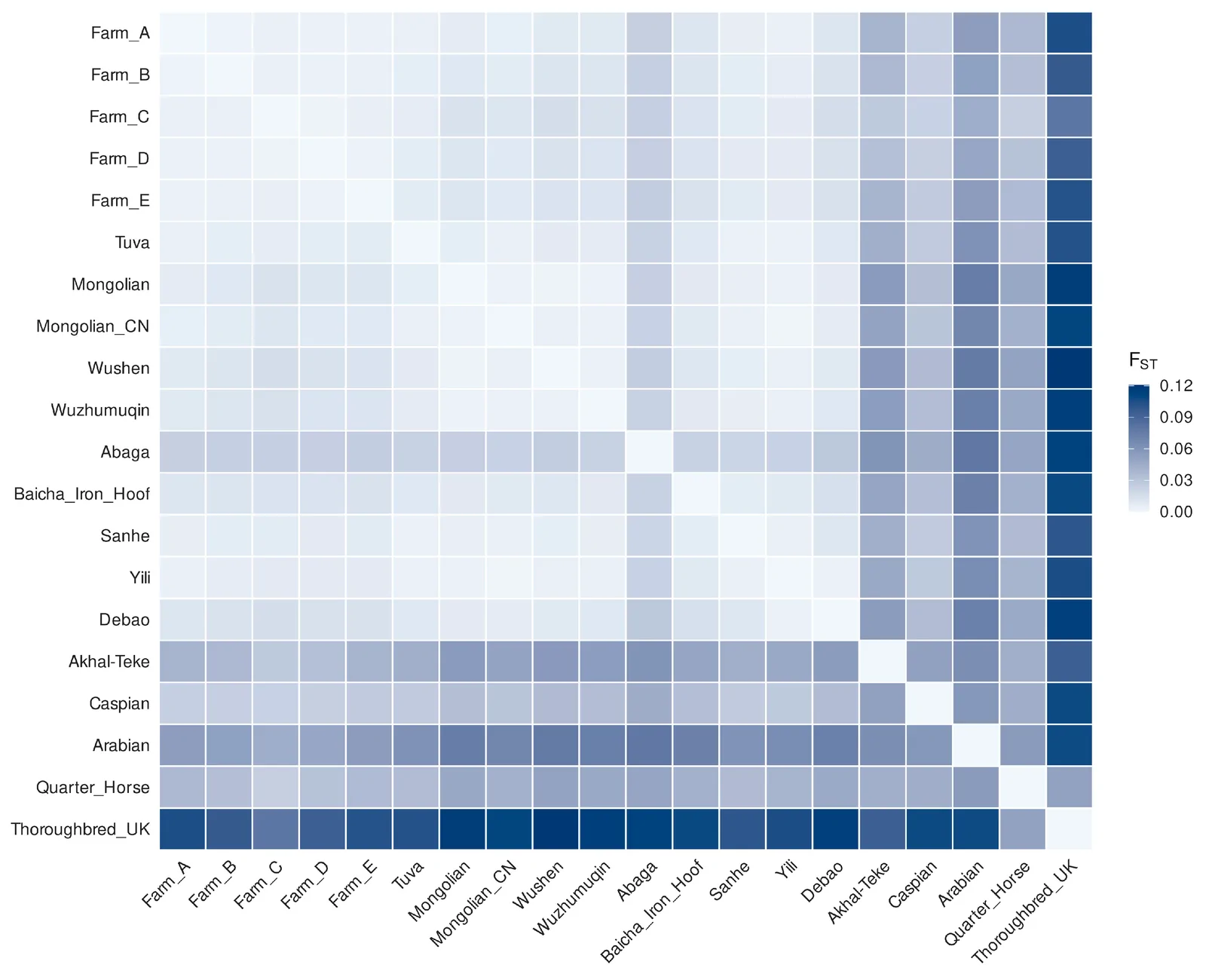
Pairwise *F*_ST_ (Weir and Cockerham) among the five Adai farms and fifteen reference breeds, on the relatedness-pruned sample. Rows and columns run from the five farms through the ten Central Asian steppe references and the three Oriental breeds to Quarter Horse and Thoroughbred; the fill runs from white at *F*_ST_ = 0 to dark blue at the largest value in the set, 0.121 between Wushen and Thoroughbred_UK.

Principal component analysis placed the horses recorded as Adai inside the Central Asian steppe cluster, and nine of the ten populations closest to them are Central Asian steppe breeds (Figure 1a and Figure 2). Pairwise *F*_ST_ is 0.0045 to Tuva, 0.0049 to Yili, 0.0056 to Sanhe and 0.0079 to Mongolian, compared to 0.1003 to Thoroughbred horses; *F*_ST_ between UK and US Thoroughbreds, two national subpopulations of one breed, is 0.0023. Between the five farms, *F*_ST_ ranges from 0.0024, between Farms C and D, to 0.0052, between Farms C and E. Each farm taken alone shows a slight excess of heterozygotes (*F*_IS_ −0.004 to −0.026), while the pooled sample shows a deficit (+0.004).

**Figure 3:**
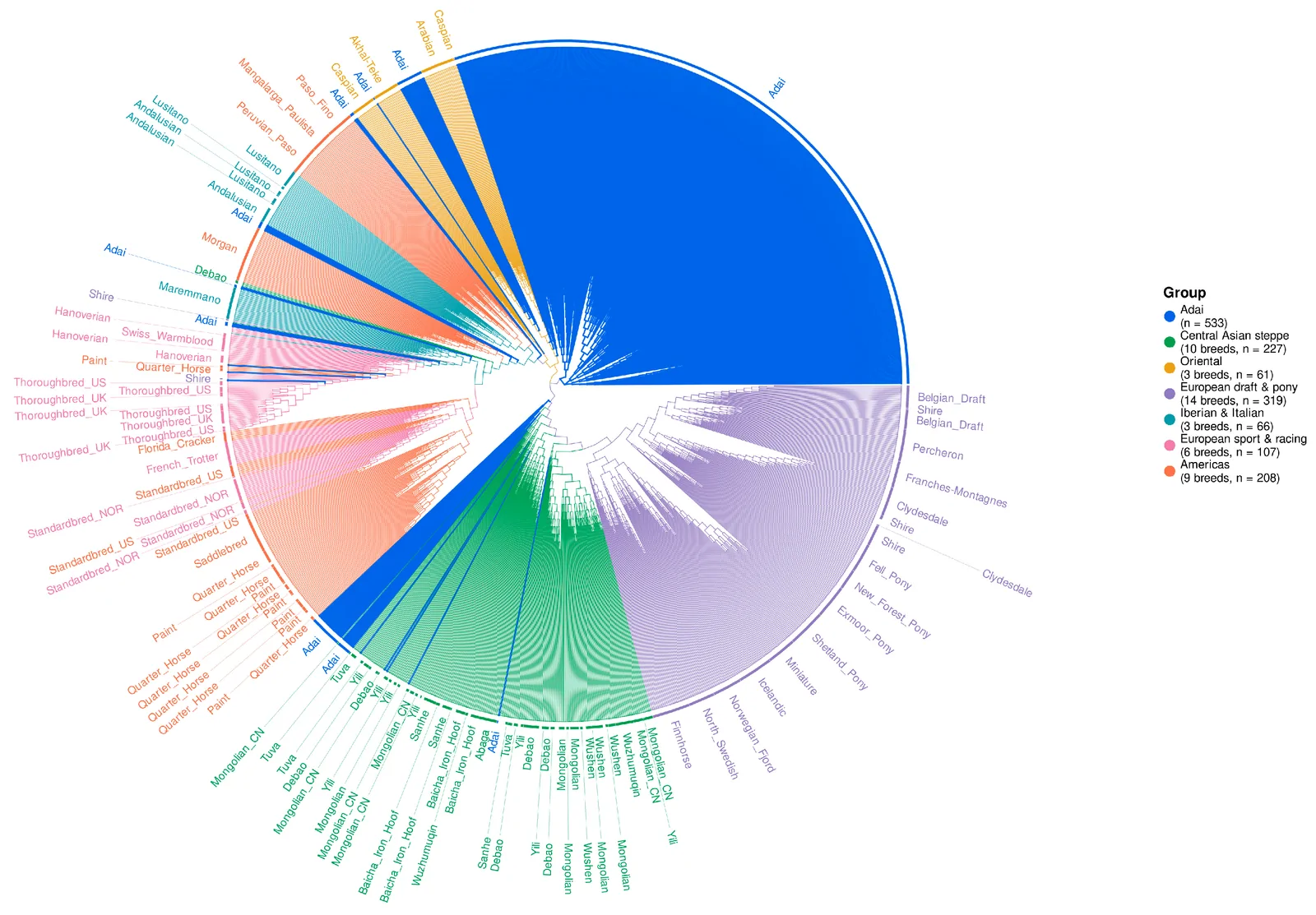
The figure shows the position of the Adai. Neighbour-joining tree of all 1521 horses in the full merged panel—988 reference horses and 533 Adai, and therefore the one analysis that retains both the seven samples later removed at --mind 0.05 and the 33 Adai without farm provenance, fitted to 1 − IBS distances on all autosomal markers and drawn as a cladogram: every branch is one unit long, and the terminal branches are stretched to a common radius, so the rim is a true circle and angular position carries topology only, not distance. Tips are coloured by regional group. Every contiguous single-breed run of tips is bracketed on the rim and named, with names placed on two radii and a hairline joining an outer label to its bracket. The same tree fitted to the provenance-filtered panel of 1481 horses places the Adai in the same region, and Section 3.7 reports the derived statistics on all three panel definitions.

Two randomly chosen Adai differ by 1 − IBS = 0.2533 and an Adai and a Mongolian horse by 0.2518, a within-to-between ratio of 1.006; the ratio also exceeds one for Tuva, Sanhe, Yili and the Chinese–Mongolian horse, and falls below it for Clydesdale (0.83) and Exmoor Pony (0.75). The individual-level tree resolves the group as one continuous block (Figure 3). Expected heterozygosity in the pooled Adai is 0.311, above every Central Asian steppe reference breed in the panel.

Weak differentiation at the level of the population does not mean individual animals are indistinguishable, and the two were tested separately. All ten pairwise *F*_ST_ values between farms have a 95% block-jackknife interval excluding zero (0.0024 to 0.0052, standard errors 1.6–4.2 × 10^−4^), so the farms are significantly differentiated from one another while being trivially so in magnitude; and the farm-to-farm range overlaps the farm-to-steppe-reference range of 0.0043 to 0.0127 (Table S13). Cross-validated individual assignment is correspondingly accurate. In an eleven-way comparison with every population subsampled to fifteen animals, overall accuracy is 67.7% against a chance rate of 9.1%, and the Adai are recovered 83.7% of the time; pairwise against each reference-steppe breed the accuracy is 86.2% to 99.9%, close to the 98.9% and 99.8% obtained against the Thoroughbred and Arabian positive controls (Tables S9–S11). Three controls locate what that measures. Restricting the Adai to the 51 relatedness-pruned animals below 5% estimated ancestry leaves accuracy essentially unchanged at 86.2% to 99.8%, so it is not an echo of the introgression. Reference breed pairs from the same published source are separated at a mean of 88.3% against 91.7% for pairs from different sources, so it is not an artefact of the genotyping platform. But the same classifier separates two herds of Adai from each other at 68.8% to 84.9%, a mean of 75.8% (Table S12). Assignment accuracy on thousands of markers therefore rises against arbitrarily small differentiation, and the herd boundary, which no one proposes is a breed boundary, behaves the same way in kind, if not in degree. The later principal components tell the same story as the first two: the Adai lie inside the steppe cloud on every pair up to PC8, and PC1 to PC10 together carry 9.8% of the variance (Table S14).

### 3.2. Thoroughbred Ancestry in Individual Horses

**Figure 4:**
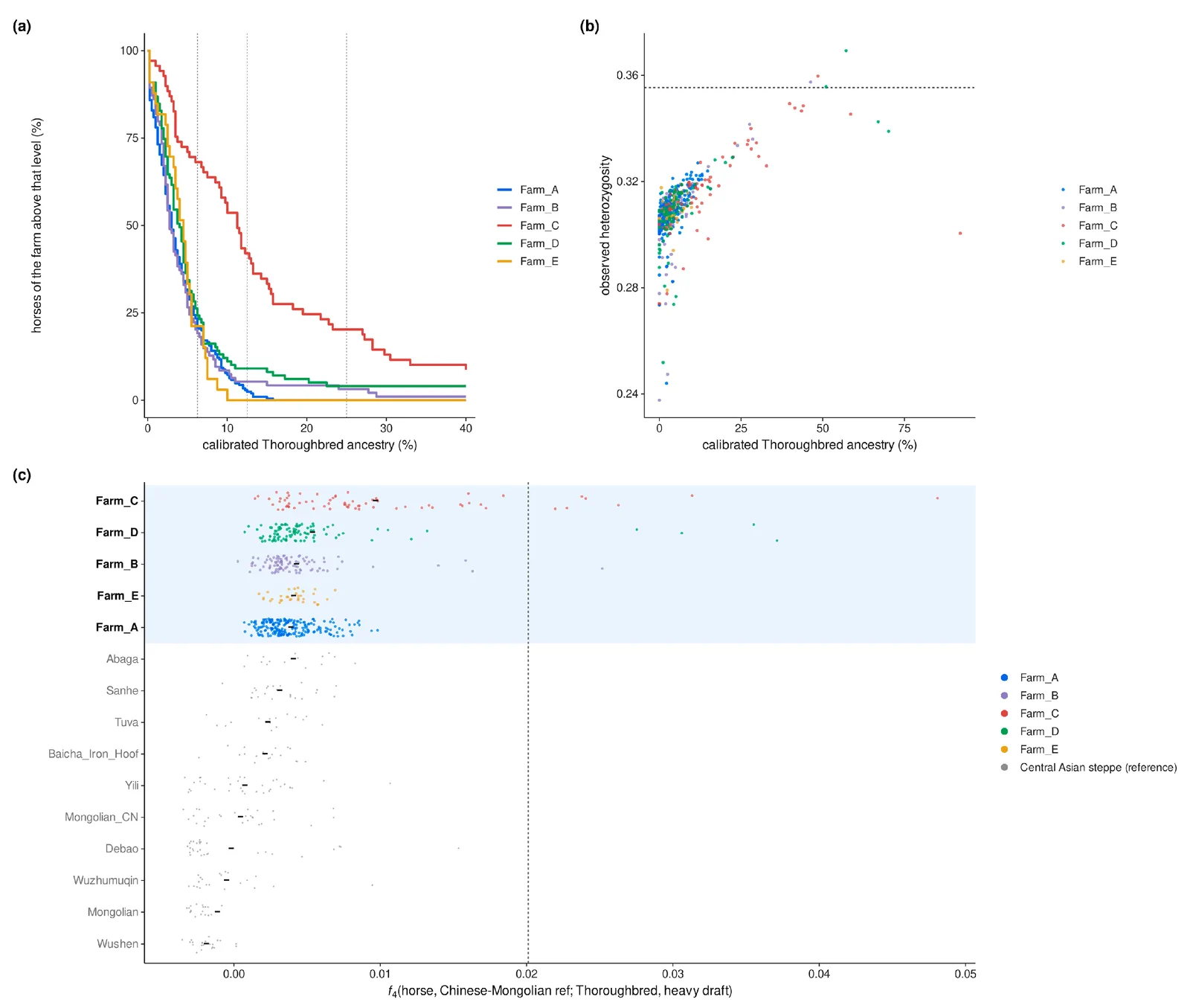
Thoroughbred introgression into horses recorded as Adai. (**a**) Share of each farm’s horses above a given level of calibrated Thoroughbred ancestry, one step line per farm; the dotted verticals mark 6.25%, 12.5% and 25%, the ancestry expected in a simple pedigree from one Thoroughbred great-great-grandparent, great-grandparent and grandparent respectively. These are expected pedigree contributions, not exact genomic proportions in any individual animal. (**b**) Observed heterozygosity against calibrated Thoroughbred ancestry, one point per Adai horse, coloured by farm; the dotted horizontal at 0.3554 is the heterozygosity predicted for an Adai × Thoroughbred F1. (**c**) *f*_4_ statistic (horse, Chinese–Mongolian steppe reference; Thoroughbred, heavy draft) for every Adai, by farm, against the same statistic for the 223 horses of the ten reference Central Asian steppe breeds (grey); the dash on each row is the population mean, and the dashed vertical at 0.0201 is the highest value reached by any reference-steppe horse. Panels a and b are on the calibrated scale of Section 3.2; panel c is the *f*_4_ statistic itself and is unaffected by the calibration.

Mean *f*_4_ (horse, Chinese–Mongolian reference; Thoroughbred, heavy draft) was 0.00507 across the 500 Adai against 0.00074 across the 223 reference Central Asian steppe horses, the highest population mean in the panel. Two criteria are reported, and they are not from the same test. The first is a position in the empirical null: 15.6% of the Adai (78 of 500) exceed its 95th percentile, and twelve animals, 2.4% of the sample, exceed its maximum (Figure 4c). The second is a fixed ancestry threshold on the *f*_4_-ratio, and for that the scale has to be calibrated. On 1800 pseudo-crosses built from real genotypes, the estimator returns α = 3.72 + 0.925 × true ancestry (*R*^2^ = 0.994, Table S3): it recovers 92.5% of the true contribution and carries a floor of 3.7 percentage points on an unadmixed Adai, so a raw α of 12.5% corresponds to a true ancestry of 9.5% and a true one-great-grandparent animal reads 15.3%. Calibrated accordingly, the pooled mean is 6.2% Thoroughbred ancestry (9.4% uncalibrated), the maximum is 92.1% (88.9%), and the share of the sample at or above one great-great-grandparent, great-grandparent and grandparent equivalent is 27.6%, 9.6% and 4.2% respectively (60.6%, 15.8% and 4.6% uncalibrated). The floor dominates the low thresholds and is negligible at the high ones, and the two null-based figures are ranks rather than values and do not move. Standard errors are available per horse (Table S1); on the α scale the median is 1.8 percentage points, with a range of 1.0 to 5.3, and the simulated replicates are, if anything, less dispersed than the jackknife implies, so the quoted intervals are conservative. Individual significance against zero is not the relevant test, since 32.7% of the reference-steppe horses themselves reach *Z* > 2 against a null mean that is positive; the empirical distribution, not a Z threshold, is what the horses are judged against.

The estimate is specific to Thoroughbred and robust to the reference. Repeating the whole cascade with an Arabian donor gives a slope of 0.259 rather than 0.925, so a genuinely half-Arabian horse reads as about 17% Thoroughbred-equivalent: the statistic responds to non-steppe ancestry generally but at roughly a quarter the rate, and α should not be read as total non-steppe ancestry (Table S3). Recomputing α under six definitions of the reference group *B*, from a single population to all ten steppe breeds, leaves the ranking of the Adai unchanged (Spearman ρ = 1.000 in every case), identifies the same twelve animals above the null maximum every time, and puts between 77 and 80 animals above the 95th percentile against 78 as published; the pooled mean does move, between 7.0% and 11.2% (Table S4). The six nearest genetic neighbours of the horse at 92.1% calibrated ancestry, across the entire panel, are six Thoroughbreds, and these are the animals displaced along PC1 towards the European sport and racing breeds (Figure 1b). Twenty horses embedded among foreign tips in both trees average 27.2% uncalibrated ancestry against 8.6% for the remainder (Wilcoxon *p* = 1.7 × 10^−4^). Thoroughbred affinity is uncorrelated with call rate (ρ = −0.015). The extreme animals were examined individually for signs of a technical origin and show none: the highest has *Z* = 20.1, a call rate of 0.9921 against an Adai median of 0.9931, and an observed heterozygosity of 0.3005, which is below the Adai mean of 0.3100 rather than above it. Contamination raises heterozygosity and carries no directional affinity; the pattern is shown by the excluded reference sample Debao-DB17 (22.5% missing, heterozygosity at the 99.7th percentile of the panel, nearest neighbour to 532 of 533 Adai); the Adai outliers show the opposite on both counts. Observed heterozygosity in the twelve averages 0.347, above the Adai mean and below the 0.3554 predicted for an Adai × Thoroughbred F1 (Figure 4b), although four animals of the 500, three of them in the twelve, sit just above that value; the ceiling is a frequency-based point estimate and these exceedances are small.

### 3.3. Unsupervised ADMIXTURE

**Figure 5:**
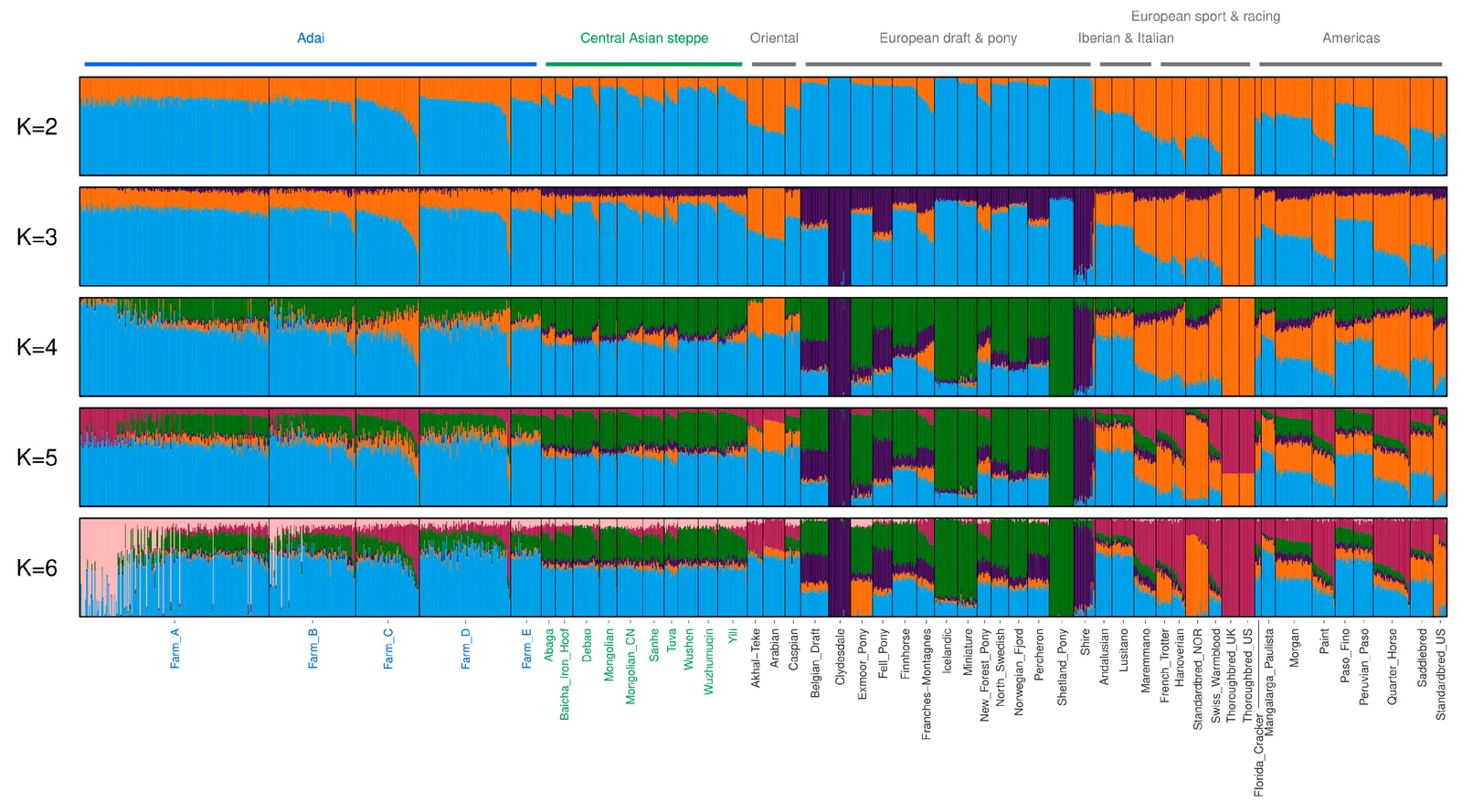
Unsupervised ADMIXTURE from *K* = 2 to *K* = 6 on the provenance-filtered farm panel (1481 horses, 21,649 LD-pruned markers). Each vertical bar is one horse, divided into the *K* inferred ancestry components; the colours distinguish components and are matched across rows for display, but carry no fixed identity. Populations are separated by white lines and labelled below, with the five Adai farms at the left; the coloured rules above the panels mark the Adai and the six regional groups of Section 2.4.

Unsupervised ADMIXTURE was run from *K* = 2 to 6 in triplicate with random seeds 1 to 3 and summarised with CLUMPAK (Figure 5). The replicates converged on a single mode at every *K*, with mean pairwise CLUMPP similarity of 1.000 at *K* = 2 to 4, 0.961 at *K* = 5 and 0.985 at *K* = 6. The range *K* = 2–6 is reported rather than a single chosen *K*, because no criterion selects one on this panel: cross-validation error falls monotonically across the range with no minimum, and Δ*K* is not usable here because the between-replicate variance in log-likelihood is effectively zero at the lower values of *K* (Table S2). This does not affect the result the introgression analysis draws from these runs, which is invariant across the range. At every *K*, the consensus contains a component fixed in Thoroughbred (mean 1.00), and the number of Adai carrying more of it than any reference-steppe horse is 13 at *K* = 2 and 12 at *K* = 3 to 6, against the twelve identified by the *f*_4_ test; two of the three replicates at *K* = 5 give 11 instead, so across all fifteen runs the count lies between 11 and 13. At *K* = 6 the mean Thoroughbred component is 0.043 in Farm A, 0.074 in Farm B, 0.075 in Farm E, 0.105 in Farm D and 0.199 in Farm C, against 0.053 across the reference-steppe horses.

### 3.4. Introgression by Farm

Thoroughbred ancestry differs across the five farms (Kruskal–Wallis χ^2^ = 63.6, df = 4, *p* = 5.1 × 10^−13^; Table 3). The test is rank-based and so is unaffected by the calibration. At Farm C, 42.0% of horses carry the calibrated equivalent of a Thoroughbred great-grandparent or more (58.0% uncalibrated), against 0.0% to 9.1% at the other four, and Farm C holds seven of the twelve animals exceeding the entire reference-steppe distribution. Farms A and E contain no such horse, and Farm E no foreign-embedded animal. The separation holds across the whole range of the statistic rather than only in its tail (Figure 4a). Because 389 first-degree pairs exist among the 500 animals and two families span more than one holding, the comparison was repeated under four schemes that do not treat the horses as independent: on the 207 relatedness-pruned animals (χ^2^ = 28.0, *p* = 1.2 × 10^−5^), on one animal drawn per family over 1000 draws (median *p* = 7.6 × 10^−7^, below 0.05 in every draw), under a linear mixed model with family as a random effect (likelihood-ratio χ^2^ = 32.0, df = 4, *p* = 1.9 × 10^−6^), and under a permutation that reassigns whole families between farms (*p* = 5.0 × 10^−4^; Table S8). The nominal *p*-value weakens by orders of magnitude once family structure is accounted for, as it should, and the farm difference survives in every case.

### 3.5. Relatedness and Inbreeding

Mean *F*_ROH_ across the 500 Adai is 0.0343, among the lowest values in the panel and below every European breed (Thoroughbred 0.211, Exmoor Pony 0.254, Clydesdale 0.284), running from 0.030 at Farm A to 0.039 at Farm C. The share of horses with at least one first-degree relative in the sample is 86.3% at Farm A, 76.6% at Farm B and 23.2% to 48.5% at the other three, and every farm exceeds 69% at second-degree or closer. Relationships also cross holdings: Farms A and B share 79 second-degree pairs, and Farms C and D and Farms D and E share first-degree pairs.

### 3.6. Body Measurements

Across all eight traits, the within-farm association with Thoroughbred ancestry is null: the largest point estimate is 0.054 standard deviations per 10 percentage points of ancestry, every 95% interval spans zero, no unadjusted *p*-value falls below 0.286, and all Holm-adjusted values are one. A horse at roughly half-Thoroughbred ancestry differs from one at the Adai mean by −0.8 to +2.3 cm in withers height, and the twelve extreme animals differ from their herdmates by +0.18 cm (SE 1.07, *p* = 0.87). The result is unchanged when age is added to the 299 animals whose year of birth is recorded: the ancestry coefficient for withers height moves from 0.074 to 0.078 cm per 10 percentage points, and every Holm-adjusted *p* remains one, even though age itself predicts live weight (*p* = 0.003), chest girth (*p* = 0.019) and cannon girth (*p* = 0.038). It is also unchanged when relatedness is accounted for, whether by refitting on one animal per family (*n* ≈ 225; all Holm-adjusted *p* ≥ 0.279) or by permuting ancestry within farm and family (*p* = 0.70 to 0.99; Tables S6 and S7).

### 3.7. Robustness

Neither of the two main results depends on which panel definition is used. Across the full merged panel of 1521 horses, the call-rate-filtered panel of 1514 and the provenance-filtered panel of 1481 on which every other analysis is run, the mean *f*_4_ of the Adai moves by less than 1.2% of its value, the share above the steppe-null 95th percentile by 0.6 percentage points, the share above the steppe-null maximum by 0.1 percentage points, the within-to-between distance ratio by 0.002 and mean *F*_ROH_ by 0.0003 (Table 4). The count of animals carrying more Thoroughbred ancestry than any reference-steppe horse is likewise stable between the two estimators: the twelve returned by the *f*_4_ test are thirteen at *K* = 2 and twelve at *K* = 3 to 6 in the ADMIXTURE consensus, and eleven to thirteen across the fifteen individual replicate runs, twelve in ten of them (Table 5).

## 4. Discussion

Ordination, pairwise differentiation and a distance tree all place the Adai inside the Central Asian steppe cluster rather than beside it (Figure 1, Figure 2 and Figure 3). Pairwise *F*_ST_ between the five farms and Yili, Tuva or Sanhe is 0.0043–0.0084 and among the farms themselves 0.0024–0.0052, an order of magnitude below the median between-breed value for horses on SNP data, 0.08–0.12 [17]; the comparable microsatellite figure, a mean of 0.08 among seven Spanish Celtic breeds [16], is of the same order but is not measured on the same kind of marker and the two are not interchangeable; both are context values from particular populations and study designs rather than universal thresholds for breed differentiation. The Adai are separated from Thoroughbred by 0.100. Two horses recorded as Adai differ from one another, on average, slightly more than an Adai differs from a Mongolian horse (within-to-between distance ratio 1.006), and the farm-to-farm and farm-to-reference ranges overlap: two herds of the same breed can be as differentiated from each other as one of them is from a Mongolian or Tuva reference group. As a population, then, the Adai is not a distinct unit. This is neither a new observation nor evidence of decline: *F*_ST_ among the six recognised Kazakh horse types is 0.0003–0.005 in 2020 animals, from which those types were proposed to be treated as a single breed [11], differentiation does not exceed 0.010 among Zhabe populations, bar one outlier [12], or 0.01 among native Xinjiang breeds [15], Kushum and Mugalzhar reach only 0.041–0.064 on microsatellites, within the range conventionally read as little differentiation [13,38], and maternal markers agree [8,14]. What the present data add is that the continuum is not interrupted at its western end: sampled at 533 animals rather than the 10 to 27 of earlier work [8,11], the Adai behaves exactly as its neighbours do. Weak differentiation is the expected condition of an extensively managed, open pastoral population, and the names applied along the continuum are geographic, functional and cultural rather than genetic. A conservation programme that treats the Adai as a genetically distinct unit to be preserved from its neighbours defends a boundary the markers do not describe, which bears on neither their adaptation to desert pasture [6,7] nor their cultural standing.

That the Adai is not a differentiated population does not mean its animals cannot be told apart from their neighbours’, and the two questions have different answers. Individual assignment against the reference-steppe breeds is accurate—83.7% in an eleven-way comparison against a chance rate of 9.1%, and 86% to 100% pairwise, so the Adai are not genomically anonymous, and a marker-based instrument is not ruled out by the weak differentiation. But the same classifier separates two herds of Adai from each other at 69% to 85%, and no one proposes that a herd is a breed. Assignment accuracy on thousands of markers accumulates from many small allele-frequency differences and rises against arbitrarily weak structure; it measures how much information the marker set carries, not whether the boundary it recovers is a breed boundary. The difference between the breed-level and herd-level accuracies is real, but it is one of degree along a continuum rather than evidence of a discrete unit. Two limits should be read with this. The comparison the breed label actually requires is the Adai against the other recognised Kazakh types: Zhabe, Kushum, Mugalzhar, Naiman and Kostanay. No genotypes of those populations are in this panel, so it could not be made here. On the evidence that those six types form a single cluster [11], it is a harder comparison. And the accuracies reported here are not the expected performance of a verification panel, which would need its own marker selection and validation.

That the population is undifferentiated does not mean it has stayed closed, and the second finding of this study is that it has not. Measured against the empirical null of 223 reference Central Asian steppe horses, 15.6% of the animals recorded as Adai exceed its 95th percentile and twelve exceed every reference-steppe horse in the panel (Figure 4). On the calibrated *f*_4_-ratio scale, 9.6% carry the ancestry expected from one Thoroughbred great-grandparent and 4.2% that expected from one grandparent, with a population mean of 6.2% (15.8%, 4.6% and 9.4% uncalibrated). Model-based estimates of ancestry are conditioned on the references supplied to the model, and no unadmixed Western-Kazakh genotype is publicly available; under supervised clustering on this panel, the Tuva horse, a Siberian steppe breed with no plausible Thoroughbred ancestry, scored indistinguishably from the Adai. The test was therefore built on an *f*_4_ statistic evaluated against an observed distribution [24] rather than on a clustering model. Unsupervised analysis, which assumes nothing about the completeness of the panel, recovers the same animals: at every value of *K* from 2 to 6, between 11 and 13 horses carry more of the Thoroughbred component than any reference-steppe horse, against the twelve identified by *f*_4_ (Figure 5, Table 5). This is concordance between two analyses of the same genotypes against largely overlapping reference populations, not independent validation of either, and we do not claim more from it than that the tail of the distribution does not depend on which of the two estimators is used. Simulation does supply the calibration the ratio needed. On pseudo-crosses built from real genotypes, the estimator recovers 92.5% of a true Thoroughbred contribution and adds a floor of 3.7 percentage points, so the uncalibrated figures overstate the prevalence of low-level ancestry and are close to correct at the top of the range; and it responds to an Arabian donor at roughly a quarter the rate, so the scale is specific to Thoroughbred-like ancestry rather than to non-steppe ancestry in general. Three properties of the signal are consistent with crossbreeding rather than with deep shared ancestry or technical artefact. It points specifically at Thoroughbred, the population furthest from the Adai in the panel and not one with which contact would be geographically expected; it is graded and continuous, as ancestry segregating from a few founders should be; and heterozygosity rises with it but, in the mean, stops below the Adai × Thoroughbred F1 expectation, averaging 0.347 in the twelve extreme animals against a predicted 0.355 (Figure 4b), whereas sample contamination, which mimics admixture in the first two respects, is not bounded in this way. None of these properties date the event. *f*_4_, principal components and ADMIXTURE measure affinity, not its age, and the heterozygosity argument distinguishes a crossbred from a contaminated sample without identifying the generation at which the cross occurred. The documentary record of twentieth-century stallion imports makes a recent origin the most economical reading, but it is an inference from that record rather than a result of these analyses.

The introgression is concentrated rather than diffuse. One farm of the five carries a mean calibrated ancestry of 15.3%, with 42.0% of its horses at one-great-grandparent equivalent or more and seven of the twelve extreme animals; the other four range from 3.9% to 6.8%, with 0.0% to 9.1% at that threshold, and two contain no horse outside the reference-steppe range at all (χ^2^ = 63.6, df = 4, *p* = 5.1 × 10^−13^, and *p* ≤ 5.0 × 10^−4^ under every treatment of the family structure). The mechanism is documented: Kazakh horses were improved by Thoroughbred, Orlov Trotter and Don stallions in the late twentieth century [20], and more than 400 stallions of specialised meat breeds entered the Mangystau and Atyrau regions between 1960 and 1987 [6], although the breeding method prescribed for the type is pure breeding with improvement by selection within it [21]. Thoroughbred outliers among Kazakh horses attested as purebred have been reported twice [11,12]; here they are quantified at the level of the individual animal and located among holdings.

Set beside genomic work on other horse populations, the contribution here is an ancestry estimate per animal in a population with no pedigrees to check it against, and the limits of that contribution follow from the same fact. Capomaccio et al. [39] characterised the Italian equine gene pool on a multi-breed panel, using ADMIXTURE for structure and MDS and TreeMix for gene flow, and documented Thoroughbred contributions to several local populations; that design establishes which populations have received gene flow, which is the question answered here at the level of the holding rather than the breed. Gmel et al. [40] went further for the Franches-Montagnes, comparing individual genomic admixture against documented historical crosses and recorded pedigrees, which is the validation the present estimates lack. That it is unavailable to us is not an oversight but the condition the study describes: in a system where mares foal on pasture and stallions hold harems, there is no pedigree against which to calibrate, and establishing one is precisely what a verification scheme would have to create. Tang et al. [15] provide the nearest geographical comparison, in native Xinjiang breeds immediately across the border, and place the Adai in a Central Asian context of similarly weak differentiation. They do not, however, bear on the calibration of the *f*_4_-ratio used here. The three together separate what this study does into the two it performs and the one it cannot.

Neither instrument by which breed membership is presently established can resolve this ancestry. Across eight body measurements in 500 horses, it showed no detectable association with any trait once farm and sex were accounted for, and none when age or relatedness was accounted for in addition. The largest point estimate was 0.054 standard deviations per 10 percentage points of ancestry; the confidence intervals exclude a difference of more than about 2.5 cm at the withers between a half-Thoroughbred animal and a typical Adai, and the twelve extreme animals, at a mean of 55.0% calibrated ancestry, differ from their own herdmates by 0.18 cm (*p* = 0.87). This is an absence of association for these eight traits under these models, not evidence that ancestry is without phenotypic consequence in general. No measure of speed, endurance, milk or meat yield was available to us. A horse carrying one Thoroughbred great-grandparent, raised on the same pasture as its contemporaries, cannot be picked out by inspection or by measuring tape, and such animals are the majority of what was detected. Nor is the signal visible in aggregate. The population is overwhelmingly of steppe ancestry, with *F*_ROH_ at 0.034 among the lowest values in the panel, and detection requires individual ancestry to be estimated against a reference distribution, which no holding can do on its own stock. The husbandry system leaves no record to trace such animals by either, since mares foal on pasture and stallions hold harems rather than being assigned to individual matings [2], so that parentage is inferred from herd composition and one stallion of outside origin leaves descendants that are neither documented nor recognisable two generations later.

The two findings carry different consequences. The absence of differentiation bears on conservation listing, since genetic-resource programmes and DAD-IS presuppose that a listed population is what its records say. The introgression bears instead on registration and trade. Animals are sold as Adai at Adai prices, and pedigree status carries a subsidy of up to 50% of the purchase price of a breeding stallion. That ceiling is set by Order of the Minister of Agriculture No. 108 of 15 March 2019 [41] rather than by the Law on Livestock Breeding [42], and neither instrument requires genetic verification. A genomic panel is a prospect rather than a result: none was developed or validated in this study. Discriminant SNP subsets assign horses of 37 other breeds at 93% accuracy, and only at the most stringent threshold [5,19]; SNP parentage verification is established practice, though no minimum marker set has been standardised [43]. Neither shows how a panel would perform in the Adai, and parentage and ancestry estimation are separate objectives. What these data add is Figure 4a, which attaches a quantified cost to each candidate threshold, and the concentration of the signal: two of the five holdings contain no animal above the reference-steppe range, so verification would not fall equally on all of them.

## 5. Conclusions

The 533 horses recorded as Adai are, in the great majority, steppe horses. They are outbred, diverse, and no more differentiated from the Mongolian and Chinese steppe breeds their name is supposed to distinguish them from than two of their own herds are from each other. That indistinguishability is shared with every one of their neighbours and is not a problem to be solved. What is a problem is that within the same population 15.6% of animals carry more Thoroughbred allele sharing than 95% of reference-steppe horses, twelve carry more than any of them, and 9.6% carry the calibrated equivalent of a Thoroughbred great-grandparent or more, and that none of it can be seen in the animal. Across eight body measurements, ancestry predicts nothing.

The problem is also not evenly spread, and that is the most useful thing we found. One farm of five accounts for most of it, two contain no horse outside the reference-steppe range at all, and the share of animals carrying the calibrated equivalent of a Thoroughbred great-grandparent or more runs from 0% to 42% across the five. This is not the condition of a breed; it is the consequence of particular decisions on particular farms, made and unmade one stallion at a time, and recorded nowhere. The Adai does not need to be protected from its neighbours. It needs to be able to be verified, and the distribution across these five holdings suggests there would be something worth verifying.

## Figures and Tables

**Table 1:** Adai sample provenance by farm.

Farm	*n*
Farm A	205
Farm B	94
Farm C	69
Farm D	99
Farm E	33

**Table 2:** Reference panel composition by source.

Source	Horses	Breeds	Array	Build
Petersen et al. [19]	795	37	SNP50	EquCab2
Kader et al. [27]	93	3 (Debao, Yili, Mongolian_CN)	SNP70	EquCab2
Han et al., EVA PRJEB55177 [28]	100	5 Chinese–Mongolian breeds	Axiom 670K	EquCab3

**Table 3:** Thoroughbred introgression by farm, on the calibrated ancestry scale of Section 3.2, with the uncalibrated mean given for comparison. The two null-based counts are ranks and are unaffected by the calibration.

	Farm A	Farm B	Farm C	Farm D	Farm E
horses	205	94	69	99	33
mean calibrated ancestry	3.9%	4.7%	15.3%	6.8%	4.2%
mean, uncalibrated	7.2%	7.9%	17.9%	9.9%	7.5%
median calibrated	3.0%	2.7%	11.2%	3.9%	4.3%
share ≥ 1/8, calibrated	2.4%	5.3%	42.0%	9.1%	0.0%
share ≥ 1/4, calibrated	0.0%	3.2%	20.3%	4.0%	0.0%
highest individual, calibrated	15.6%	46.3%	92.1%	70.2%	9.8%
above the steppe-null 95th pct	18	8	39	12	1
above the steppe-null maximum	0	1	7	4	0
foreign-embedded (both trees)	1.5%	2.1%	15.9%	4.0%	0.0%

**Table 4:** Robustness of the main results across panel definitions.

Claim	Full Panel (1521)	Call-Rate-Filtered (1514)	Provenance-Filtered (1481)
Adai mean *f*_4_	0.00506	0.00501	0.00507
Adai above steppe-null 95th pct	15.0%	15.0%	15.6%
Adai above steppe-null maximum	2.3%	2.3%	2.4%
within/between distance ratio	1.008	1.006	1.006
Adai *F*_ROH_	0.0346	0.0346	0.0343

**Table 5:** Agreement between the *f*_4_ test and ADMIXTURE.

Claim	*f*_4_ Test	ADMIXTURE Consensus	ADMIXTURE, 15 Replicate Runs
Adai above every reference-steppe horse	12	13 at *K* = 2, 12 at *K* = 3–6	11–13; 12 in ten of the fifteen

## Data Availability

Reference genotypes are public: Petersen et al. [19], Kader et al. [27] (Dryad https://doi.org/10.5061/dryad.357cr) and Han et al. (European Variation Archive, PRJEB55177) [28]. Adai genotypes: https://doi.org/10.5281/zenodo.22748191.

## Supplementary Materials

The following supporting information can be downloaded at: https://www.mdpi.com/article/10.3390/genes17101292/s1. Table S1: *f*4 and Thoroughbred-equivalent ancestry per Adai horse, with standard errors; Table S2: ADMIXTURE model selection; Table S3: Calibration of the *f*4-ratio on simulated crosses; Table S4: Sensitivity of α to the composition of the reference group B; Table S5: Jackknife of the ratio against the numerator alone, per horse; Table S6: Phenotype models with age; Table S7: Phenotype relatedness sensitivity; Table S8: Farm comparison under four treatments of family structure; Table S9: Multiclass assignment accuracy; Table S10: Assignment confusion matrix; Table S11: Pairwise assignment accuracy; Table S12: Assignment controls; Table S13: *F*ST with block-jackknife intervals; Table S14: PCA eigenvalues PC1–PC10.

## Abbreviations

The following abbreviations are used in this manuscript:

*F*ST	Fixation index
*F*ROH	Inbreeding coefficient from runs of homozygosity
IBS	Identity by state
LD	Linkage disequilibrium
NJ	Neighbour joining
PCA	Principal component analysis
ROH	Runs of homozygosity
SNP	Single-nucleotide polymorphism
